# Highlights from the seventh European Multidisciplinary Colorectal Cancer Congress (EMCCC) 2014

**DOI:** 10.3332/ecancer.2015.497

**Published:** 2015-01-06

**Authors:** Cornelis JA Punt, Iris D Nagtegaal, Cornelis J van de Velde, Regina Beets-Tan, Annemieke Cats, Nicoline Hoogerbrugge, Corrie A Marijnen

**Affiliations:** 1 Department of Medical Oncology, Academic Medical Centre, Amsterdam 1105AZ, The Netherlands; 2 Department of Pathology, Radboud University Medical Centre, Nijmegen 6525GA, The Netherlands; 3 Department of Surgery, Leiden University Medical Centre, Leiden 2333ZA, The Netherlands; 4 Department of Radiology, Maastricht University Medical Centre, Maastricht 6229HX, The Netherlands; 5 Department of Gastroenterology, Antoni van Leeuwenhoekziekenhuis, Amsterdam 1066CX, The Netherlands; 6 Department of Human Genetics, Radboud University Medical Centre, Nijmegen 6525GA, The Netherlands; 7 Department of Clinical Radiotherapy, Leiden University Medical Centre, Leiden 2333ZA, The Netherlands

**Keywords:** Dutch Colorectal Cancer Group, DCCG, European Multidisciplinary Colorectal Cancer Congress, EMCCC, colorectal cancer

## Abstract

It is widely recognised that colorectal cancer has become a complex disease and involves many medical disciplines. The mission of the European Multidisciplinary Colorectal Cancer Congress (EMCCC), which is an initiative of the Dutch Colorectal Cancer Group (DCCG), is to facilitate the interaction between relevant disciplines on current issues of research. The three-day meeting in Amsterdam in November 2014 assembled approximately 450 participants with nine different disciplines from 38 countries worldwide. On day one, workshops on imaging, surgery, medical oncology/pathology, radiotherapy, and genetics were followed by the keynote lecture of the congress. On day two and three, a total of 35 established international opinion leaders presented lectures in plenary sessions on prevention and screening of early colorectal cancer, genetics, translational research, biomarkers, organ-saving treatment in rectal cancer, current controversies, and multidisciplinary management. Posters from submitted abstracts were displayed, with selected abstracts being orally presented.

## Introduction

The primary aim of the EMCCC, which was organised on 23–25 November in Amsterdam for the seventh time by the DCCG, is to bring together key opinion leaders from all disciplines relevant to the diagnosis, treatment, and research of colorectal cancer (CRC), to share their opinions and recent data available, and to facilitate a stimulating interaction between representatives of these disciplines. Here we present a conference report that highlights the topics that were discussed and presented during this outstanding meeting.

## Workshops

Workshops on the following topics were held. *Imaging*: MR—what you have always wanted to know; *Surgery*: integration of MRI, anatomy, and surgery; colorectal cancer auditing; *Medical Oncology/Pathology*: tumour profiling for selection of high-risk patients; *Radiotherapy*: elderly patients with rectal cancer; and *Genetics*: up to date in hereditary cancer in 2014.

## Keynote lecture

N Arber, Israel, presented a great overview on the current status of chemoprevention of CRC. He concluded that chemoprevention together with early detection and lifestyle/nutrition are among the key elements to reduce the prevalence of CRC, and that specifically the effect of low-dose aspirin is usually underestimated. Gene variants should enable us to better predict its effects.

## Prevention and screening

S Halloran, UK, and E Dekker, The Netherlands, discussed the current status and lessons that were learned from the UK and Dutch screening programmes, respectively. Halloran predicted that faecal immunochemical testing (FIT) was to remain the standard screening test for the next 15 years, and Dekker presented data on the first six months of the Dutch screening programme, which showed higher than expected participation and positivity rates. A study on the comparison of two FIT’s is ongoing. P Laird, USA, explained the clinical use of DNA methylation markers for early detection and prediction of chemoresistance, and showed that CpG island methylator phenotype-high (CIMP-H) tumours encompass most BRAF mutant tumours and that CIMP-H is responsible for most sporadic microsatellite instabiligy (MSI)-high tumours by MLH-1 promotor methylation. M van Leerdam, The Netherlands, presented guidelines for surveillance colonoscopies in patients with adenomas and CRC, and argued that the risk for metachronous high-risk adenomas/CRC should be determined by a risk score based on adenoma characteristics.

## Early colorectal cancer

A Repici, Italy, advocated the use of morphological criteria for endoscopic submucosal dissection (ESD), and concluded that ESD is still underperformed and associated with poor outcome in Western countries. P Quirke, UK, addressed the pathobiology of early CRC, and identified quantitative measurements (area of submucosal invasion and width of invasive carcinoma) as risk factors in pT1 tumours. Lastly, N Mortensen, UK, discussed the selection of patients for local excision, which included oncological and functional criteria as well as patients’ choice. He explained that for T1 tumours, the results of local excision were not as good as tumour microenvironment (TME), T2 and nodal disease required definitely more than local excision, but that in an ageing population, dysfunction after anterior resection and aversion to a colostomy were all the driving forces to search for better alternatives.

## Genetics

Hereditary mixed polyposis syndrome, caused by an unusual mutation and with different GREM1 upstream duplications, and polymerase proofreading polyposis were discussed by I Tomlinson, UK. He concluded that an identical phenotype can result from very different defects. Then, H Thomas, UK, explained that familial colorectal cancer (FCC) is a heterogeneous group, and that surveillance should be initiated around age 30–40 years. He provided arguments for the timing of follow-up surveillance depends on the risk groups, and then presented data from a pooled prospective analysis on this topic from six European centres. N Hoogerbrugge, The Netherlands, stressed the need for improvement on the recognition of high hereditary risk for CRC, and proposed a strategy to find all hereditary CRC.

## Translational research

I de Vries, The Netherlands, presented data on the feasibility, safety, and immune responses upon vaccination with MSI peptides-loaded dendritic cells of patients with Lynch syndrome. She discussed her ongoing clinical protocol of vaccination for Lynch patients with MSI tumours prior to surgery. These were with autologous dendritic cells loaded with two HLA class I hereditary nonpolyposis colorectal cancer (HNPCC) and carcinoembryonic antigen (CEA) peptides in combination with keyhole limpet hemocyanin (KLH). Antigen-specific immune responses were observed, and no serious adverse events were noted. T Bishop, UK, discussed chemoprevention in high-risk populations and follow-up data on the CAPP2 study, in which carriers of hereditary cancer are randomised between supplemental aspirin and placebo. At 12 years, 45/434 participants in the placebo group, and 25/427 in the aspirin group developed Lynch syndrome cancers. He presented unpublished data on the relationship between Lynch syndrome and obesity in this study, showing no effect for obesity in participants treated with aspirin. Reynolds, UK, reviewed vessel co-option as a mechanism of resistance to angiogenesis inhibitors, and presented novel data on the correlation between pathological growth patterns and intrinsic/acquired resistance to anti-angiogenic treatment. He also explained that a desmoplastic growth pattern is responsive, but a replacement growth pattern is associated with intrinsic and acquired resistance. This latter growth pattern is therefore an important therapeutic target, with targeting the tumour invasion as a potential strategy.

## Oral abstract presentations

The following selected abstracts were presented. D Saraste, Sweden, compared screening versus non-screening detected colorectal cancer, and its impact on preoperative workup and treatment. She concluded that screening results in a significantly higher percentage of early cancer detection, however the intensity of treatment was comparable between the two groups; A Sie, The Netherlands, found that a fourfold-increased detection of Lynch syndrome by raising age limit for tumour genetic testing on mismatch-repair deficiency from 50 to 70 years is cost-effective; A Boleij, USA, tested the hypothesis that mucosal exposure to bacteroides fragilis toxin (BFT) gene is required for a direct impact on carcinogenesis in humans, and found that BFT is highly prevalent in the colon mucosa of colorectal cancer patients; and A Appelt, Denmark, presented data on watchful waiting, and a national Danish study of chemoradiotherapy (CRT) as definitive treatment of low rectal cancer. She concluded that the use of high-dose radiotherapy allowed for a high proportion of distal rectal cancers to be treated with CRT alone, and that more than 50% of patients could be spared a colostomy.

## Biomarkers

G Siravegna, Italy, addressed the topic of liquid biopsies for the monitoring of colorectal cancer treatment, and discussed the molecular heterogeneity of cancer, the clonal evolution in cells, and the genotyping of CRC in peripheral blood. Unpublished data were presented on the appearance of adenomatous polyposis coli (APC) and Kristen Ras (KRAS) mutations in circulating DNA as early predictors of acquired resistance to cetuximab treatment in patients. JP Medema, The Netherlands, presented data of his own group on the molecular subtyping of stage II colon cancers, as well as the recent consensus on molecular subtypes by the CRC subtype consortium—SAGE bionetworks. This consensus has resulted in four different prognostic molecular subtypes: the MSI immune, canonical, metabolic, and mesenchymal subtype. R Salazar, Spain, discussed novel data of the biology of RAS and BRAF mutations and the implications for clinical practice by showing recent results of signal transduction inhibition in preclinical and clinical models. New studies in patients with RAS mutated tumours employ MEK inhibitors with either ERBB inhibitors or IGF1R inhibitors, and in patients with BRAF mutated tumours employ BRAF/MEK inhibitors. Finally, A Lugli, Switzerland, advocated the use of intratumoural budding as an independent adverse prognostic factor, and explained its pathogenetic, clinical, surgical, and pathologic aspects.

## Organ-saving treatment in rectal cancer

Local excision after chemoradiotherapy and ongoing trials on this topic were discussed by C Coco, Italy. He discussed that a clinical complete response (CR) is not easy to define and previous results show that this predicts a pathological CR in a range between 25% to 90%. C Marijnen, The Netherlands, proposed an algorithm based on clinical staging for the use of external beam radiotherapy and brachytherapy in situations of planned surgery versus organ preservation. Issues in the selection of patients for a wait-and-see policy, and the role of imaging in this process, were discussed by G Beets, The Netherlands. Data from his own studies show that wait-and-see is feasible in a small proportion of patients after CRT, and local regrowth was observed in 12% of patients at two years, which was usually easily manageable. This topic was continued by G Brown, UK, who presented data on the value of serial MRI assessment of tumour regression. Employing serial MRI monitoring demonstrates that tumours continue to show regression, with 75% of patients reaching a maximum response at six months, and that this allows a greater rate of recruitment of initially advanced cancers for deferral of surgery.

## Controversies

The following topics were the subject of Oxford-style debating:

1. Irresectable liver-only metastases should be treated with minimal invasive modalities, and not systemically,
  - *Pro:* T Helmberger, Germany: efficacy has been demonstrated, modalities should be incorporated into multidisciplinary concepts;
  - *Con:* C Punt, The Netherlands: individual non-randomised trials have used inappropriate historical controls, randomised trials are underpowered, systematic analyses have not shown any benefit;
2. MSI should be assessed in all patients with colorectal carcinomas,
  - *Pro:* I Frayling, UK: Lynch syndrome is found in all age categories, testing is easy, safe, simple, and inexpensive;
  - *Con:* M Ligtenberg, The Netherlands: if threshold for testing is set at age 70 years, and all high-risk stage II patients are tested: 40% of patients do not need a test which avoids costs, counselling, and distress;
3. Colorectal surgery—robotic surgery is preferred over laparoscopy,
  - *Pro:* D Jayne, UK: current benefits of robotic surgery are real (if marginal in terms of patient outcome), costs are relative and will reduce, and provides an evolving platform that will enhance surgical performance;
  - *Con:* P Tanis, The Netherlands: robotic surgery has no proven benefit, investments should be made in further implementing evidence-based conventional laparoscopic surgery rather than promoting unproven expensive technology);
4. Adjuvant chemotherapy is standard in high-risk rectal cancer,
  - *Pro:* A Cervantes, Spain: data from pooled analyses and subgroups show its benefit;
  - *Con:* K Bujko, Poland: benefit of adjuvant chemotherapy is unproven, but its harm to patients is.

## Multidisciplinary management

A Martling, Sweden, discussed the timing of radiotherapy and surgery in rectal cancer, with data from the ongoing Stockholm III trial, which showed low toxicity, tumour downstaging, and logistical benefits for short course radiation therapy (RT) with delayed surgery and a tendency for more postoperative complications for short course RT followed by immediate surgery. She highlighted the promising results of the downstaging effects and logistic benefits of short-course radiotherapy with delayed surgery. An update on trials with intensified neoadjuvant regimens in rectal cancer was given by C Rödel, Germany. He argued that, given the current results, such regimens should primarily be aimed at the reduction of distant metastases or the improvement of organ preservation. M Koopman, The Netherlands, compared the design and outcomes of recent trials on systemic maintenance treatment in metastatic CRC. She concluded that superiority is preferred over non-inferiority designs, and that current data support the use of capecitabine plus bevacizumab in this setting. A Vahrmeijer, The Netherlands, presented promising results of ongoing research on image-guided surgery, and he identified the lack of standardised contrast agents as an important hurdle to bring this modality to clinical practice. R Fijneman, The Netherlands, summarised the current status and future directions of biomarkers for metastatic disease. He explained the added value of individual prognostic biomarkers, and supported the functional characterisation of individual recurrent breakpoint genes to further define their role in cancer biology and clinical behavior. A de Haes, The Netherlands, addressed the importance of patient empowerment and shared decision-making. Without the latter, she warned that evidence-based medicine could turn into evidence-based tyranny.

## Conclusion

The seventh EMCCC meeting in Amsterdam highlighted the current status and novel data on a wide spectrum of issues which aim to advance the process of diagnosis and treatment of CRC. Multidisciplinary interaction was guaranteed by the plenary presentations of all subject categories. Developments in screening, early detection, genetics, treatment strategies in early and advanced rectal cancer, and the development of new biomarkers as well as biomarker-driven treatment strategies were the most prominent topics of the programme. During the session on biomarkers, the president of the Dutch Colorectal Patient Organisation (SPKS) presented the first patient-information leaflet on the value of DNA testing for the selection of treatment in metastatic CRC. Mutual understanding across many disciplines who are involved in CRC, and sharing of each other’s breakthroughs, complexity, and pitfalls are essential for achieving progress in this very common disease, and for this the EMCCC again provided an excellent platform.

